# Study on large deformation of soil–rock mixed slope based on GPU accelerated material point method

**DOI:** 10.1038/s41598-024-57362-x

**Published:** 2024-03-24

**Authors:** Bingke Liu, Wen Wang, Zhigang Liu, Ningpeng Ouyang, Kejie Mao, Fuchuan Zhou

**Affiliations:** 1 School of Civil Engineering, Chongqing University, Chongqing, 400045 China; 2 Department of Engineering, Durham University, Durham, DH1 3LH UK; 3 China Construction Fourth Engineering Division Corp. LTD, No. 220 Weihai Road, Panyu District, Guangzhou, 511400 China; 4 Hehai College, Chongqing Jiaotong University, Chongqing, 400074 China; 5 Chongqing Vocational Institute of Engineering, Chongqing, 402260 China

**Keywords:** Material point strength reduction method, Soil–rock mixture, Slope stability, Stone content, Large deformation, GPU-based MPM, Civil engineering, Natural hazards

## Abstract

This study assesses the effect of stone content on the stability of soil–rock mixture slopes and the dynamics of ensuing large displacement landslides using a material point strength reduction method. This method evaluates structural stability by incrementally decreasing material strength parameters. The author created four distinct soil–rock mixture slope models with varying stone contents yet consistent stone size distributions through digital image processing. The initial conditions were established by linearly ramping up the gravity in fixed proportionate steps until the full value was attained. Stability was monitored until a sudden shift in displacement marked the onset of instability. Upon destabilization, the author employed the material point method to reconstruct the landslide dynamics. Due to the substantial computational requirements, the author developed a high-performance GPU-based framework for the material point method, prioritizing the parallelization of the MPM algorithm and the optimization of data structures and memory allocation to exploit GPU parallel processing capabilities. Our results demonstrate a clear positive correlation between stone content and slope stability; increasing stone content from 10 to 20% improved the safety factor from 1.9 to 2.4, and further increments to 30% and 40% ensured comprehensive stability. This study not only sheds light on slope stability and the mechanics of landslides but also underscores the effectiveness of GPU-accelerated methods in handling complex geotechnical simulations.

## Introduction

**Figure 1:**
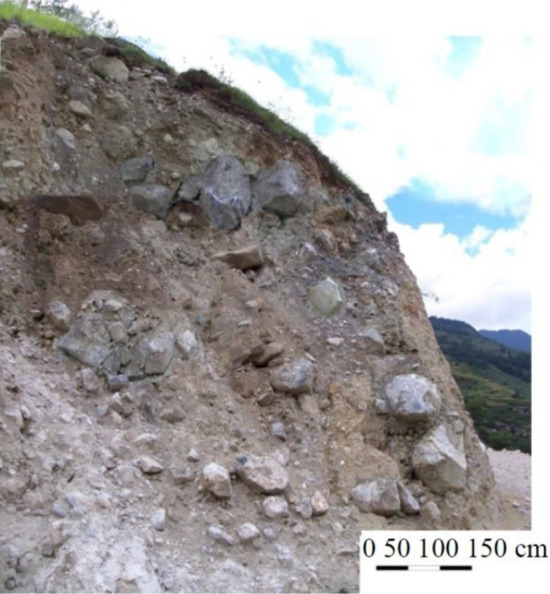
A cross-section of an SRM slope (Wen-Jie et al. 2008).

Soil–rock mixture (SRM) slope is a typical anisotropic slope that commonly exists in nature, which is composed of high-strength block and low-strength soil and shows extreme anisotropy^1–3^. In the Three Gorges reservoir area of the Yangtze River in China, many SRM slopes can be found^4,5^, as shown in Fig. 1. Trying to avoid such slopes in engineering practice is impossible, so analyzing the stability of SRM slopes and the large displacement landslides happening after destabilization is necessary^6–11^.

In the past decades, the limit equilibrium method (LBM) has been widely used to study the stability of slopes, especially homogeneous soil slopes^12,13^, but for SRM slopes, this method is almost invalid. The main difference between the two materials composing SRM slopes is that the connection strength between soil particles is much less than the cementation strength between the minerals composing the blocks^1,14^. The deformation and damage characteristics of the soil material are determined by the filling and compacting of the voids inside the soil and the relative misalignment and slip generated between the particles, while the damage of the crushed boulders is the process of compacting the microfractures, compressing the pores, and expanding and penetrating the crack sprouts^15,16^. The physicomechanical properties of SRM constituent materials are highly variable, ranging from fine earth particles to large fragments, in addition to the interlocking adhesion between the two. In order to be able to investigate the properties of SRM materials, a series of research works have been carried out by many scholars in recent years.

As for experimental research, Duo et al.^17^ conducted a series of large triaxial tests, and based on these, they studied the mechanical properties of SRM using fractal theory. Yang et al. proposed the concept of critical stone content on the basis of a large number of shear tests on soil–rock mixtures in the Three Gorges reservoir area, and they considered the stone content rate as a key factor controlling the overall stability of SRM^18^. Xu et al. conducted in situ large horizontal push shear tests in the natural state and obtained reliable stress–strain curves^19^. In terms of numerical simulation as well as theoretical research, for SRM materials, the difficulty is the model setup, and there are two main technical means to construct the most realistic SRM model. One is the digital image processing technique^4,20^, which obtains images through image acquisition techniques such as photography or CT scanning, and then processes the digital images to build a digital model. Another effective method is the random generation method, which puts rock block patterns with a certain size, distribution, and content into a specific area one by one and quickly generates multiple SRM models^21^. Xu et al. used digital image processing techniques to establish a soil–rock mixture model and conducted stability studies using the finite element square intensity reduction method to investigate the shear zone distribution characteristics and stability coefficients of SRM slopes. Zhao et al. used the latter method to establish a soil–rock mixture slope that can consider any shape, content, and distribution and explored the effects of stone content rate and bulk distribution on the stability of SRM slopes.

The role of numerical simulation in modern scientific research is becoming more and more indispensable, and it has become an important research tool following theoretical analysis and physical experiments^22^. In recent years, a series of numerical methods have been proposed and developed, including methods for large and small deformations^23–26^. In this paper, the author adopts the new numerical method of the material point method (MPM), which is developed from the particle-in-cell method(PIC)^27^. Sulsky and Chen et al.^28^ changed the solution of the constitutive equations to be solved on material points, overcoming the difficulty of considering the time dependence in the PIC method. It combines the advantages of Lagrangian and Eulerian descriptions and shows sufficient advantages for the simulation and analysis of large deformation problems^29,30^. The superiority of the MPM has been realized by scholars in several fields and has been applied to several collars: aerospace^31^, explosion analysis^32,33^, high-speed collision^34^, biomechanics^35^, and geotechnical engineering^36–39^, producing some convincing results. In this paper, firstly, the author used digital image processing techniques to establish four SRM slope models containing 10%, 20%, 30%, and 40% of the rock, respectively. Next, the author investigated the safety factor of the slopes using the material point strength reduction method and explored the rock content’s influence on the slopes’ stability. Finally, the author reconstructed the whole process of landslide motion using the MPM and obtained the material motion and deformation characteristics such as slip velocity, slip distance and plastic strain of the slope body at typical moments.

## Material point model

### Methodology

**Figure 2:**
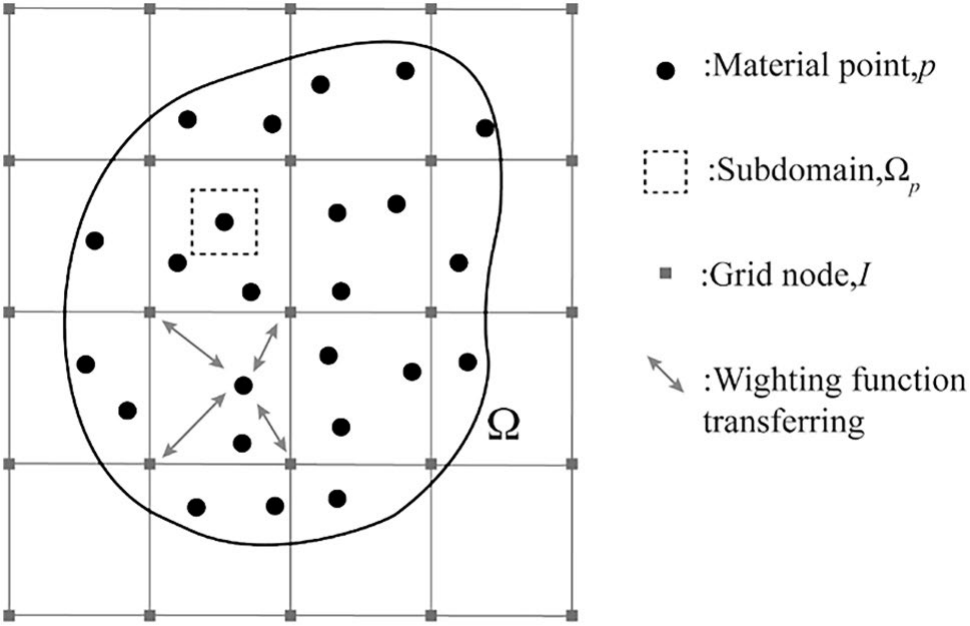
Material point method discrete format.

As shown in Fig. 2, the MPM uses two description systems, material points, and grid, to characterize the behavior of the simulated object Ω during the simulation. The simulated object Ω is discretized into a series of Lagrangian material points and moves in the background grid, and all the information is stored in the material points, including mass, momentum, energy, strain, and stress.

In this paper, the classical material point method is still used to carry out the research work because this paper requires a large amount of computation. Although the improved MPM, such as the generalized interpolation material point method(GIMP)^40^ and the B-splines material point method(BSMPM)^41^, have better stability and accuracy, these improved methods come at the cost of increased computational effort. The detailed theory of MPM, and the strong form and weak form of the governing equations are detailed in the literature^30,35,42^, and for brevity, this paper will not be expanded.

### Discretization formulations

As shown in Fig. 2, using a rectangular linear finite element background mesh for material point discretization, the density of the object can be approximated as,

1 $$\rho (x_{i} ) = \sum\limits_{p} {m_{p} \delta (x_{i} - x_{ip} )}$$

where $m_{p}$ represents the mass of the material point, $\delta$ is the Dirichlet function, and $x_{ip}$ and $x_{i}$ denote the coordinates of the material point and grid node, respectively. The equivalent integral weak form of the momentum equation and the given surface force boundary condition is

2 $$\int_{\Omega } \rho \ddot{u}_{i} \delta u_{i} dV + \int_{\Omega } \rho \sigma_{ij}^{s} \delta u_{i,j} dV - \int_{\Omega } \rho b_{i} \delta u_{i} dV - \int_{{\Gamma_{t} }} \rho \overline{t}_{i}^{s} \delta u_{i} dA = {0}$$

where $\delta u_{i}$ denotes the imaginary displacement at the boundary $\Gamma_{u}$ which is equal to 0, $\sigma_{ij}^{s} = \sigma_{ij} /\rho$ denotes the specific stress; $\overline{t}_{i}^{s} = \overline{t}_{i} /\rho$ denotes the boundary surface force; and the imaginary displacement satisfies $\delta u_{i} \in \{ \delta u_{i} |\delta u_{i} \in C^{0} ,\delta u_{i} |\Gamma_{u} = 0\}$, where $C^{0}$ denotes the set of continuous functions. According to the Eq. (2), the author can transform the weak form of the control equation into the form of summation over the masses as follows,

3 $$\sum\limits_{p} {m_{p} } \ddot{u}_{ip} \delta u_{ip} + \sum\limits_{p} {m_{p} } \sigma_{ijp}^{s} \delta u_{ip} - \sum\limits_{p} {m_{p} } b_{ip} \delta u_{ip} - \sum\limits_{p} {m_{p} } \overline{t}_{ip}^{s} h^{ - 1} \delta u_{ip} = 0$$

where the subscript p denotes the physical quantity carried by the material point at position $x_{ip}$ and $h$ is the hypothetical boundary layer thickness, introduced to convert the last term at the left end of the weak form into a volume fraction.

Within each computational step of the MPM, the relationship between the material points and the background grid is fixed until the background grid is discarded, and the information transfer between them is achieved through the interpolation of the form function $N_{I} (x_{i} )$ built on the background grid nodes. In this paper, variables on the background grid nodes are represented as quantities with a subscript $I$. The shape function $N_{I} (x_{i} )$ is

4 $$N_{Ip} = \frac{1}{{4}}(1 + \xi_{I} \xi_{p} )(1 + \eta_{I} \eta_{p} ),I = 1,2,3,{4}$$

in which, $\xi_{p} \in [ - 1,1]$, $\eta_{p} \in [ - 1,1]$ denote the natural coordinates of the material point, $(\xi_{p} ,\eta_{p} )$ is the natural coordinates of the grid nodes, the range of values is $( \pm 1, \pm 1)$.

At the beginning of each calculation step of the material point method, the weight of each material point to the surrounding grid nodes is first calculated, and the information of the material points is mapped to the grid nodes according to the weights (P2G). The mass and momentum of a grid node can be expressed as:

5 $$m_{I} = \sum\limits_{p} {m_{p} N_{Ip} } ,\;\;P_{Ii} = \sum\limits_{p} {m_{p} v_{pi} N_{Ip} }$$

where, $m_{p}$ and $v_{pi}$ denote the mass and velocity of the material points, respectively.

Using the USF format, the stresses of the material points are updated at the beginning of the calculation step. Once the grid gets the information from the material point, the author can solve for the strain rate $\dot{\varepsilon }_{ijp}^{n - 1/2}$ and the spin rate $\Omega_{ijp}^{n - 1/2}$ of the material point according to the velocity gradient of the grid nodes, so that the author can calculate the objective stress rate, i.e., the Jouman stress rate.

6 $$\dot{\varepsilon }_{ijp}^{n - 1/2} = \sum\limits_{I} \frac{1}{2} \left( {{\text{S}}_{Ip,j}^{n} v_{iI}^{n - 1/2} + S_{Ip,i}^{n - 1/2} v_{jI}^{n - 1/2} } \right)$$

7 $$\Omega_{ijp}^{n - 1/2} = \sum\limits_{I} \frac{1}{2} \left( {{\text{S}}_{Ip,j}^{n} v_{iI}^{n - 1/2} - S_{Ip,i}^{n - 1/2} v_{jI}^{n - 1/2} } \right)$$

8 $$\dot{\sigma }_{ij} = \sigma_{ij}^{\nabla } + \sigma_{ik} \Omega_{jk} + \sigma_{jk} \Omega_{ik}$$

where $\sigma_{ij}^{\nabla } = C_{ijkl}^{\sigma J} \dot{\varepsilon }_{kl}$ is Jouman stress rate, $C_{ijkl}^{\sigma J}$ is the elastic stiffness tensor. Once the objective stress rate is obtained, the author can compute the trial stress $\tilde{\sigma }_{ijp}^{n} = \sigma_{ijp}^{n - 1} + \dot{\sigma }_{ijp}^{n - 1/2} \Delta t$ using an isotropic elastic model. Using the return mapping algorithm, the trial stress is brought to the Drucker-Prager criterion, and the stress beyond the yield surface is pulled back to the yield surface to obtain the true stress $\sigma_{ijp}^{n}$. Then, the author can calculate the internal forces of the grid nodes based on the obtained stress information and apply external forces according to the boundary conditions. In this paper, only gravity is considered for external forces, and after obtaining the information on nodal forces, the momentum equation can be solved on the grid nodes as follows.

9 $$f_{iI}^{int} = - \sum\limits_{p} {S_{Ip,j} } \sigma_{ijp} \frac{{m_{p} }}{{\rho_{p} }}$$

10 $$f_{iI}^{ext} = \sum\limits_{p} {S_{Ip} } m_{p} b_{ip} + \sum\limits_{p} {S_{Ip} } \frac{{m_{p} }}{{\rho_{p} }}\overline{t}_{ip} h^{ - 1} \frac{{m_{p} }}{{\rho_{p} }}$$

where $\sigma_{ijp}$ denotes the Cauchy stress of the material points, $N_{Ip,j}$ denotes the derivatives of shape functions and $\rho_{p}$ denotes the density of the material points, where $b_{ip}$ denotes the body force, such as gravity, $\overline{t}_{i}^{s} = \overline{t}_{i} /\rho$, represents the boundary surface force and $h$ represents the boundary layer thickness.

11 $$p_{iI}^{n + 1/2} = p_{iI}^{n - 1/2} + f_{iI}^{n} \Delta t$$

where, $f_{iI}^{{}} = f_{iI}^{ext} + f_{iI}^{{\text{int}}}$, superscript represents the time step, and $p_{iI}^{n + 1/2}$ represents the resultant force of node force at the nth time step.

The last sub-step is to remap the updated momentum back to the material point (G2P) and update the position of the material point. In order to make the calculation more stable, the author use a hybrid momentum mapping format, i.e., a mixed format of PIC and FLIP, for mapping the momentum information to reduce the instability of FLIP^43^.

12 $$v_{ip}^{n + 1/2} = \alpha \sum\limits_{I} {P_{iI}^{n + 1/2} N_{Ip}^{n} /m_{I}^{n} + (1 - } \alpha )\left( {v_{ip}^{n - 1/2} + \Delta t\sum\limits_{I} {\frac{{f_{Ii} N_{Ip} }}{{m_{I} }}} } \right)$$

where the first term denotes PIC format and the second term denotes FLIP format. In this paper, $\alpha = 0.02$. In addition, one point to note is that the momentum information of the PIC mapping is the momentum of the grid nodes after updating, and the momentum information of the FLIP mapping is the momentum increment in the change time step. In addition, the PIC format maps the updated momentum of the grid nodes, and FLIP is the increment of momentum within the time step. The positions of the material points are updated as follows:

13 $$x_{ip}^{n + 1} = x_{ip}^{n} + \Delta tv_{ip}^{n + 1}$$

where $x_{ip}^{n}$ and $x_{ip}^{n + 1}$ denote the location of material points at n and (n + 1) time step, respectively.

## MPM implementation under a GPU architecture

**Figure 3:**
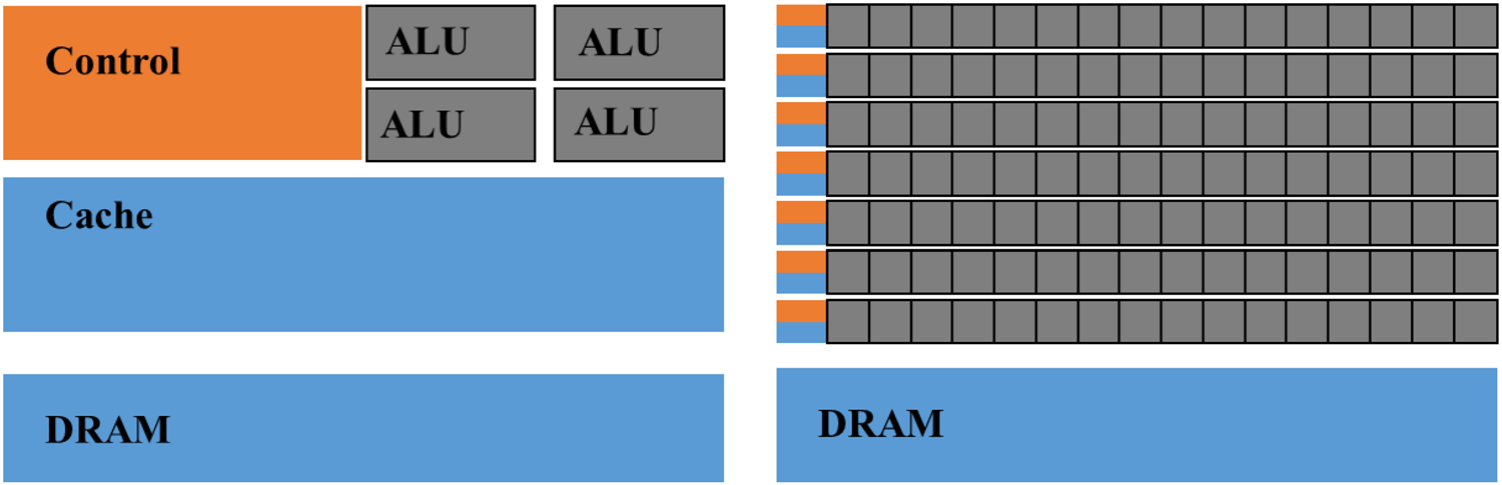
Schematic diagram illustrating the chip architecture of Central Processing Units (CPUs) and Graphics Processing Units (GPUs). The latter is comprised of thousands (or even more) Arithmetic Logic Units (ALUs). CPU architectures are primarily dedicated to control units and cache memory, allowing for significant reduction in physical space allocated to ALUs compared to GPU architectures.

Given the extensive computational analysis involved in our research, the existing open-source MPM codes predominantly support CPU parallel execution^30^. Despite the improved computational efficiency, the computation time remains unacceptably high when dealing with large-scale calculations. To address this issue, the author is contemplating the development of our computational program on the GPU, as depicted in Fig. 3, leveraging the primary architectural differences between CPUs and GPUs.

On GPU chips, the majority of physical space is dedicated to Arithmetic Logic Units (ALUs), whereas in CPUs, most of the physical space is allocated to chip host scheduling and control microsystems. Compared to CPUs, GPUs feature a higher number of cores, lower thread scheduling costs, and higher memory bandwidth. GPUs have a hierarchical structure, with threads as the smallest computing units organized into thread blocks that form a hierarchical grid. GPUs typically launch thousands (or even more) threads that execute the same instructions in parallel, enabling massive parallelism. Additionally, modern GPUs offer high throughput with peak memory bandwidth approaching terabytes per second. Contemporary GPU architectures, including Ampere, Turing, and Volta, provide a computational framework that is well-suited for the locality of the material point method from a high-performance computing perspective. Due to the particle nature of the MPM method in conjunction with the presence of the Eulerian background grid, MPM does not require complex neighbor search algorithms like other meshless methods such as SPH^44–46^, nor does it necessitate intricate data structures for storing grid data like grid-based methods such as PFEM^47^, which are not as GPU-friendly. For intense and non-local computations (i.e., the bidirectional mapping between background grid nodes and material points), the author employs direct atomic operations (scatter–gather paradigm).

**Figure 4:**
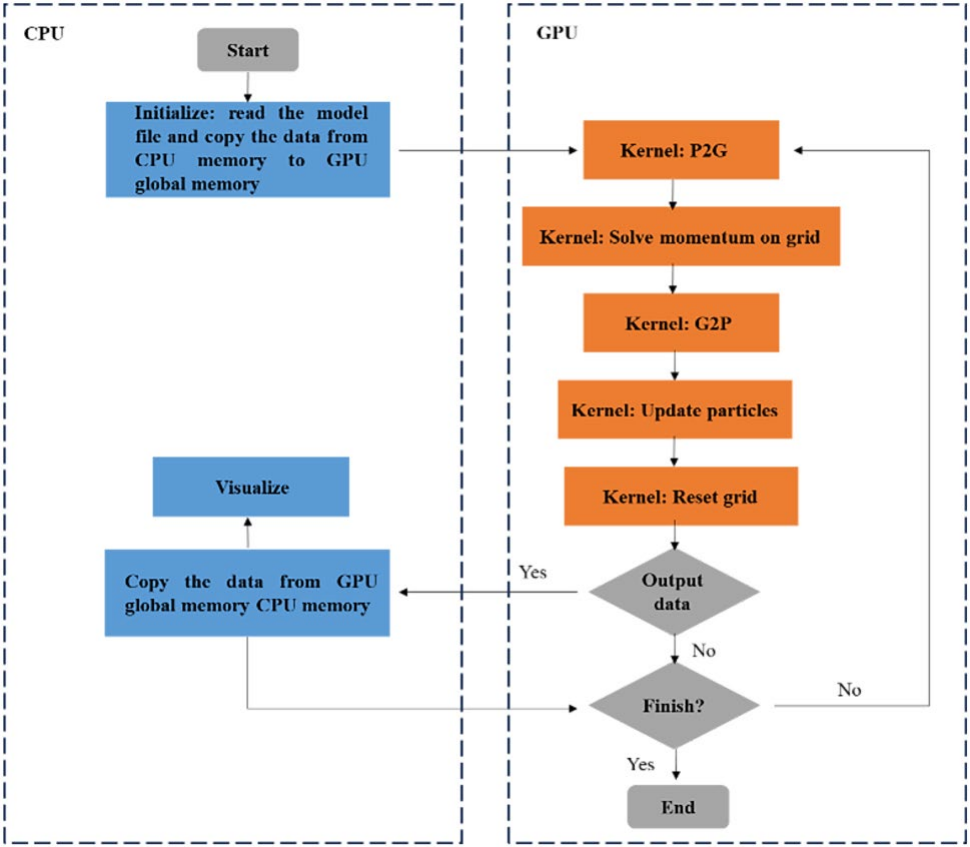
Algorithm flowchart of our GPU-based MPM.

Figure 4 presents the algorithmic workflow of our high-performance GPU-based MPM. The program is divided into two parts, with the left side representing the control flow executed on the CPU backend. It includes model initialization, data transfers between CPU memory and GPU memory, and other related operations. The right side represents the computation part executed on the GPU backend, involving the transfer of particle information to the grid (P2G), solving the momentum equations, transferring the updated information back to the material points (G2P), and updating the material point states.

## Model setup and material parameters

### Image-based modeling method

**Figure 5:**
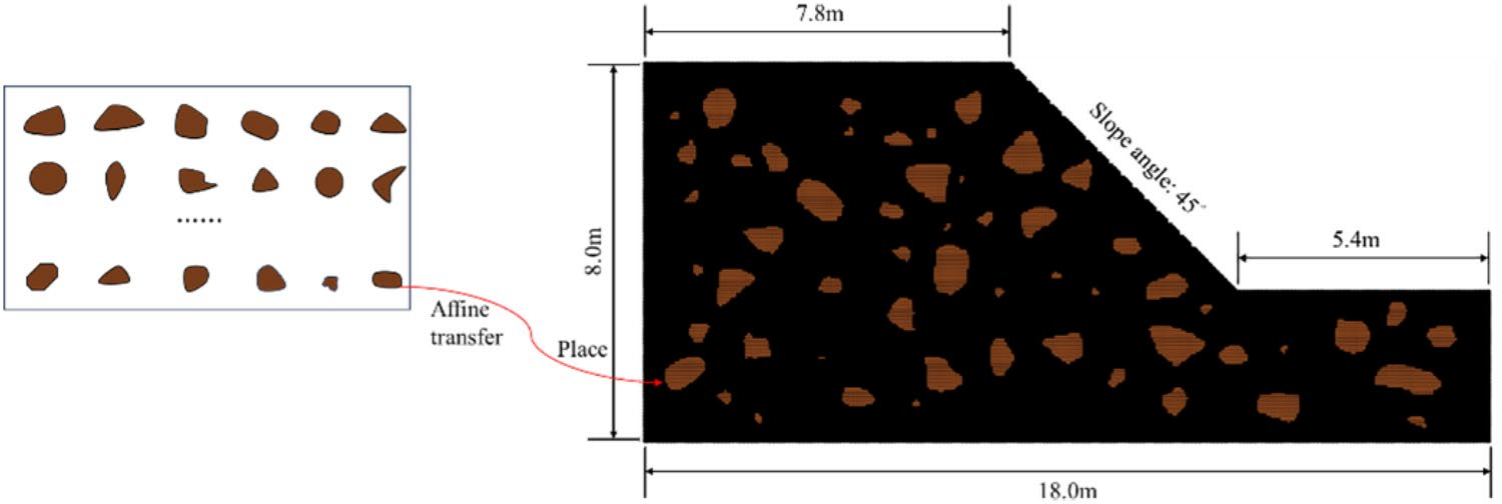
Schematic of the generation of the MPM model of a soil–rock mixture slope using the image processing techniques.

Before establishing the material point model, the author utilizes the PyGran library to generate a series of particles with varying shapes, as shown in the left subplot of Fig. 5. PyGran is a Python library specifically designed for Discrete Element Method (DEM) simulations, providing functionalities for generating DEM particle shapes. It supports the generation of spherical, cubic, and polyhedral particles, among other shapes, along with flexible parameter controls and visualization tools. After generating a particle library, the author employs image processing techniques to convert the images into matrices and apply affine transformations. Subsequently, the author places these transformed particles into our slope model, as illustrated in the right subplot of Fig. 5. Using this technique, the author generates slope models with different stone contents of 10%, 20%, 30%, and 50%. Finally, the author performs a binary thresholding process on the model images to differentiate between stones and soil, labeling them accordingly and mapping the pixel positions to the material point locations. The position information and corresponding particle IDs are stored, and following the algorithmic workflow depicted in Fig. 4, the model is initialized and subjected to computational analysis. In the setup of our model, a background grid spacing of 0.1 m was selected, and four material points were allocated within each grid cell, resulting in a dense simulation network comprising a total of 42,497 material points.

All simulations in this study are conducted on a PC equipped with an Intel® Core™ i7-12700F CPU @ 2.10 GHz and an NVIDIA® GeForce RTX™ 3080 with 10GB GPU. To achieve higher numerical accuracy during our computations, the author opted for double-precision floating-point numbers for all numerical operations.

### Material parameters

The material parameters are shown in Table 1 .

**Table 1:** Mechanical parameters of the SRM slope.

Parameters	Soil	Stone
$\rho /\left({\text{kg}}/{{\text{m}}}^{3}\right)$	1800	2410
$E/{\text{MPa}}$	50	20,000
$\nu$	0.35	0.2
$\phi /^\circ$	24	42
$c/{\text{kPa}}$	10	900
$\psi /^\circ$	24	42

**Figure 6:**
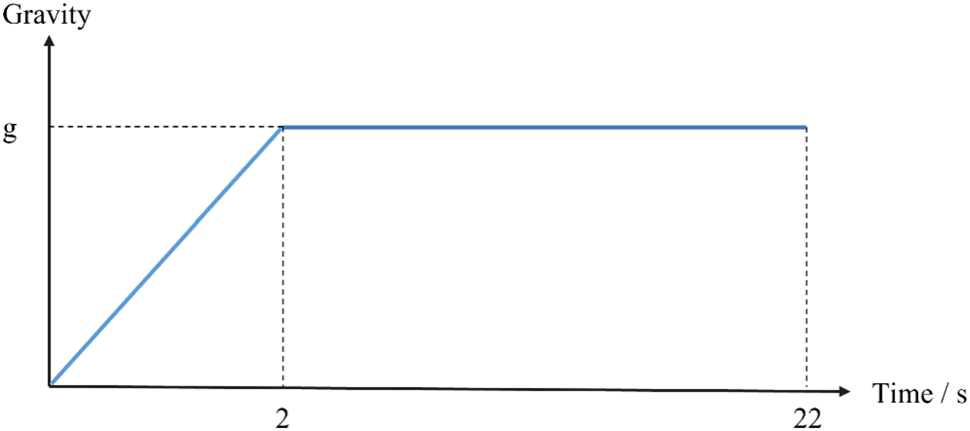
Increment of gravity over the simulation period.

As shown in Fig. 6, the total duration of the simulation is 22 s. The first 2 s will be loaded linearly with gravity to obtain a stable initial state of the model, followed by discounting the material parameters until the slope is damaged. To make the simulation more stable, this paper sets a small-time step, 0.01ms.

## Stability analysis of the soil–rock mixture slopes

### Stability of slopes

In this paper, the reduced material strength can be expressed as:

14 $$c^{\prime} = \frac{c}{srf}$$

15 $$\phi^{\prime} = \arctan \frac{\tan \phi }{{srf}}$$

where $c$ and $c^{\prime}$ denote cohesion and reduced cohesion, respectively, $\phi$ and $\phi^{\prime}$ denote internal friction angle and reduced internal friction angle, respectively, and srf reparents the reduction factor, which varies from 1 and increases by 0.1 each time until damage occurs on the slope.

**Figure 7:**
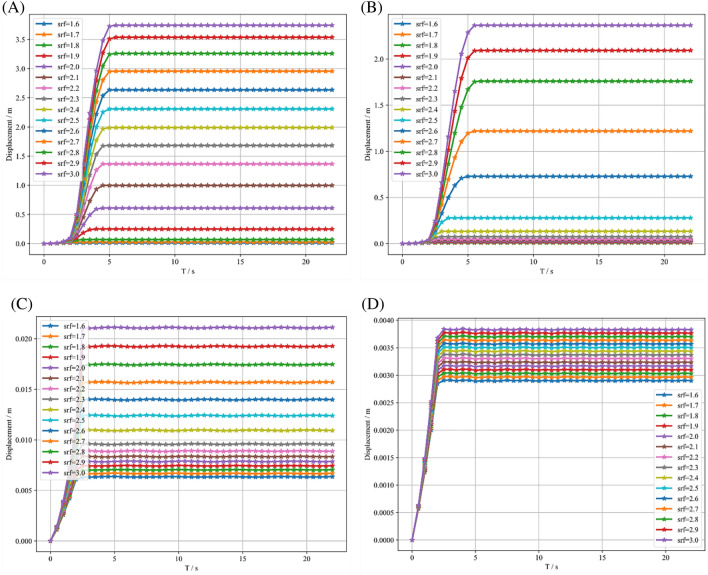
Maximum displacement variation with time for mixed soil–rock slopes with different reduction factors. (**A**) Maximum displacement variation curve of SRM slope with 10% rock. (**B**) Maximum displacement variation curve of SRM slope with 20% rock. (**C**) Maximum displacement variation curve of SRM slope with 30% rock. (**D**) Maximum displacement variation curve of SRM slope with 40% rock.

In this paper, the reduction factor starts from 1.6 and takes an increasing linear mode with a 0.1 increase each time, and the maximum reduction factor is 3.0. During the simulation, the maximum displacements of the four SRM slope models were recorded, and their variation with time was also plotted as shown in Fig. 7, where (A), (B), (C), (D) represent the maximum displacement variation of slopes with rock content of 10%, 20%, 30%, and 40%, respectively. The onset of instability can be determined by observing whether there is a sudden change in the vertical displacement of the slope crest. This approach intuitively reflects changes in the slope stability state and provides a clear quantitative criterion for assessing its safety.

According to Fig. 7, when the reduction factor is equal to 1.9 and 2.4, the displacement of slopes with 10% and 20% of stone content undergoes abrupt shifts. In other words, their safety factors are 1.9 and 2.4, respectively. For slopes with stone content of 30% and 40%, the maximum displacement is 0.1% and 0.02% of the slope length, and in this paper, it is considered that the slope is still in a stable state without damage at this time. These indicate that the growth of stone content is beneficial to maintain the stability of the slope. In terms of the magnitude of the values, the biggest difference between two models of smaller stone content and two models of larger stone content is that the values of the first two are at the meter level, and the last two are at the millimeter level. In terms of the trend of the curves, the first two have an additional straight segment than the latter two. These are due to the fact that the majority of the displacements in the latter two come from the settlement caused by gravity application rather than landslides caused by strength reduction.

In addition, the two slope models with small rock content have one identical feature. They have the same trend of displacement variation, and both consist of a settlement phase during gravity loading, a phase of rapid displacement growth, and a stabilization phase. The displacements quickly increase to a maximum value under the corresponding discount factor and then remain constant. In other words, the large displacement landslide after the instability of the soil–rock mixed slope is completed in a short time. With the increase of the reduction factor, the time for the slope to recover stability also grows. The SRM slope with 10% rock content takes at most 5 s to restabilize, and the SRM slope with 20% rock content restabilizes at t = 5.5 s when srf > 2.7.

**Figure 8:**
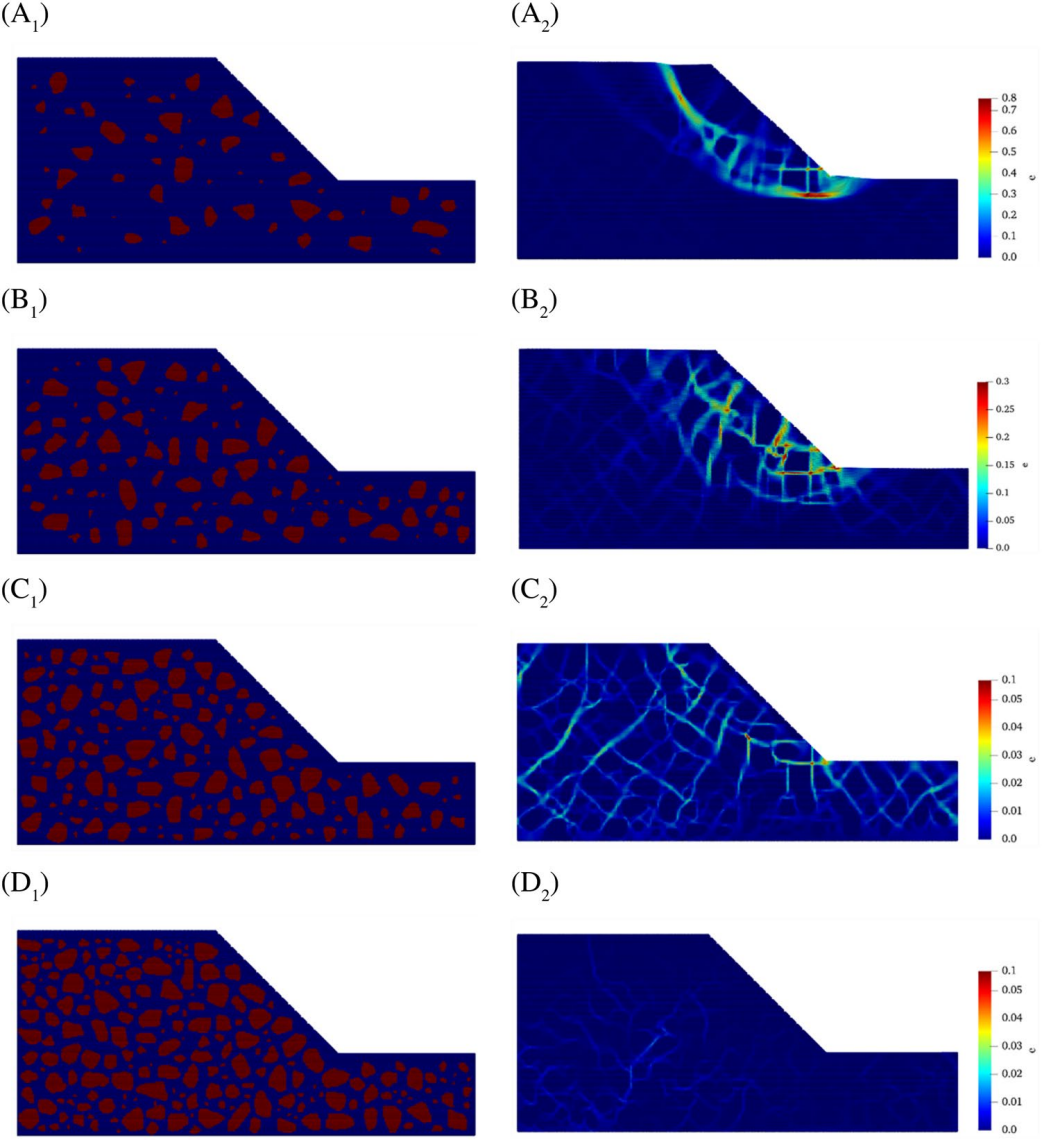
Model and corresponding stability analysis results of 4 SRM slopes. (**A1**,**A2**) Slope model with 10% rock content and shear zone distribution at instability. (**B1**,**B2**) Slope model with 20% rock content and shear zone distribution at instability. (**C1**,**C2**) Slope model with 30% rock content and shear zone distribution at instability. (**D1**,**D2**) Slope model with 40% rock content and shear zone distribution at instability.

To enable the study of damage patterns of SRM slopes, the shear zone clouds of these four models at their corresponding safety factors or maximum reduction factors were plotted, as shown in Fig. 8.

According to Fig. 8, overall, the shear zone distribution of the SRM slope has a distinct rock-wrapping characteristic as well as a staggered distribution. This is different from the one major through-shear zone formed by the pure soil slope when it is unstable. The similarity between the damage patterns of SRM slopes and pure soil slopes is that both types of slopes accumulate large plastic deformation at the foot of the slope. In addition, the shear deformation gradually decreases as the rock content increases, and after the rock content reaches 40%, the shear deformation is distributed only in the interior of the slope, and the values are small.

According to Fig. 8A1,A2, with the stone content equal to 10%, a major shear zone is formed from the foot of the slope to the top of the slope when the slope is unstable, and the shape is approximately circular, which is similar to that of the pure soil slope. It indicates that the level of anisotropy of the SRM slope is low when the rock content is low. Unlike the pure soil slope, there are several interlaced shear zones developed around the main shear zone. In addition, there is a shear zone behind the main shear zone with small plastic deformation. Compared with Fig. 8A1, it can be found that this shear zone is located exactly at the position with small or no rocks. According to Fig. 8B1,B2, for the SRM slope with 20% rock, there is not a major shear zone formed in the slope but a series of interlaced shear zones at instability. This indicates that the slope anisotropy level is high at this time. Near the slope toe, the plastic deformation is concentrated, and it is also the place where the shear zone is most densely distributed. According to Fig. 8C1,C2,D1,D2, for SRM slopes with stone content over 20%, it is known from the previous analysis that these two slopes do not experience instability in the calculation, and the paper gives the distribution of their shear zones when the reduction factor is equal to 3.0.

**Figure 9:**
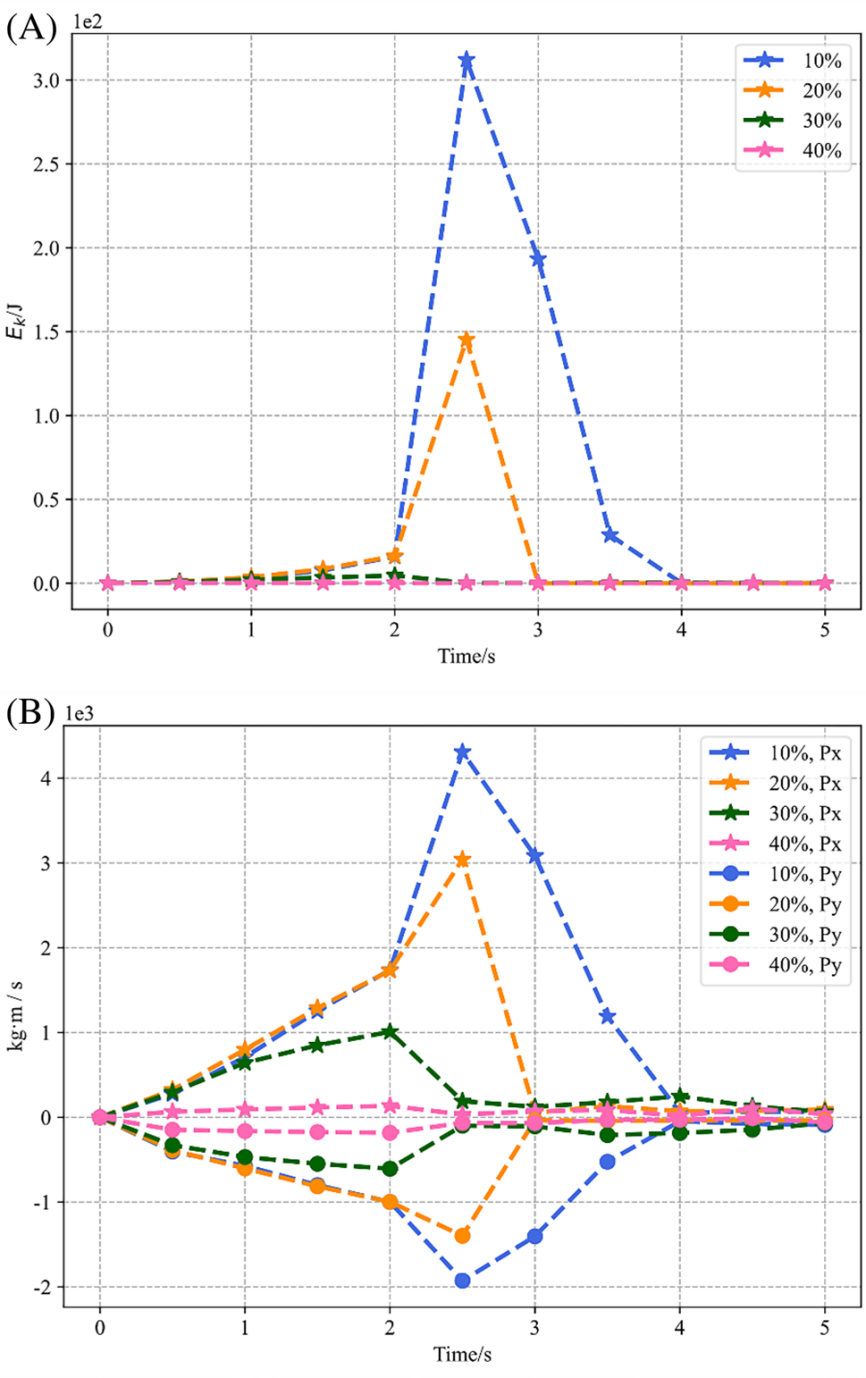
Kinetic energy and momentum variation of four SRM slope models. (**A**) Kinetic energy variations. (**B**) Momentum variations.

To investigate the dynamics of these four slope models during instability or at the maximum reduction factor, the kinetic energy and momentum of the models are monitored, as shown in Fig. 9.

According to Fig. 9A, The most drastic change in kinetic energy is found in the slope with the smallest stone content, followed by the slope with stone content equal to 20%, and the other two models have almost no fluctuation in kinetic energy. Both slopes with drastic kinetic energy variation reach their peak kinetic energy at 2.5 s, but their kinetic energy returns to zero at different times, and the model with smaller stone content is delayed by 1.0 s. In other words, the duration of the landslide of the slope with 10% rock is longer than that of the slope with 20% rock by 1.0 s. According to Fig. 9B, the movement pattern of the landslide is dominated by horizontal motion, and the changing trend of momentum is kept almost the same with kinetic energy.

### Development of slope failure

In order to explore the further development of slope damage as the reduction factor increases, and considering that the two slope models with rock content over 20% change insignificantly during the study, this section discusses in detail the further damage of slopes with rock content equal to 10% and 20%, respectively.

**Figure 10:**
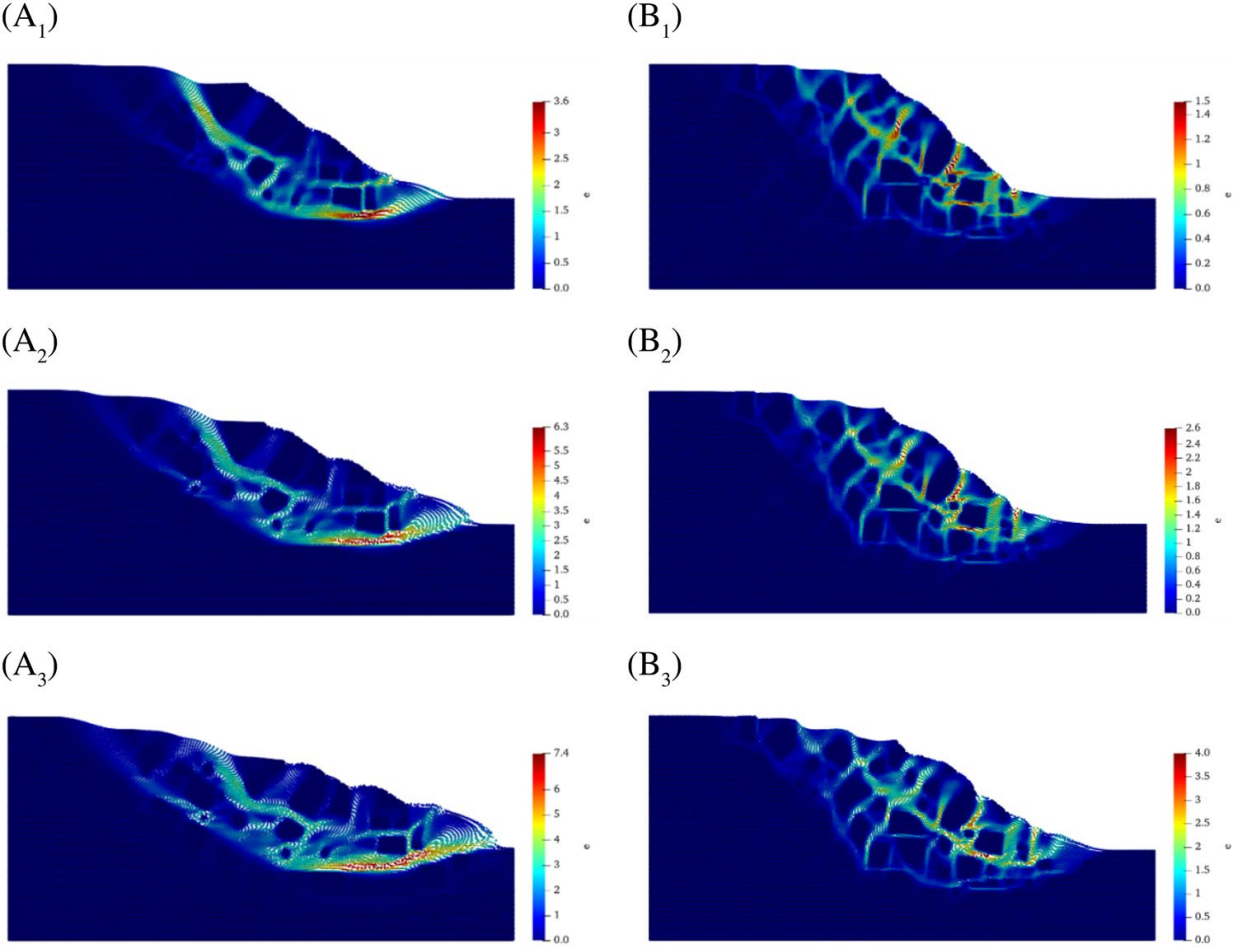
Extension of the shear zone in SRM slopes as the reduction factor increases. (**A1**–**A3**) Shear zone distribution of SRM slope with 10% rock content at the reduction factor equal to 2.2, 2.5 and 2.8, respectively. (**B1**–**B3**) Shear zone distribution of SRM slope with 20% rock content at the reduction factor equal to 2.6, 2.7 and 2.8, respectively.

Figure 10 gives snapshots of the further development of the shear zone with increasing reduction factor for both slopes. Figure 10A1–A3 gives the distribution of shear zones in the SRM slope with 10% rock content when the reduction factor equals 2.2, 2.5, and 2.8, respectively, and Fig. 10B1–B3 gives the distribution of shear zones in the SRM slope with 20% rock content when the reduction factor equals 2.6, 2.7 and 2.8, respectively. The damage mode of the former is similar to arc sliding, and it forms two main shear zones, which are approximately parallel and approximate to arcs. The latter also forms two major shear zones as the reduction factor increases, and they are formed by a series of small shear zones distributed around the stone, showing obvious anisotropy. The common feature of both is that the shear zone range does not increase as the reduction factor increases, but only the plastic strain accumulates.

**Figure 11:**
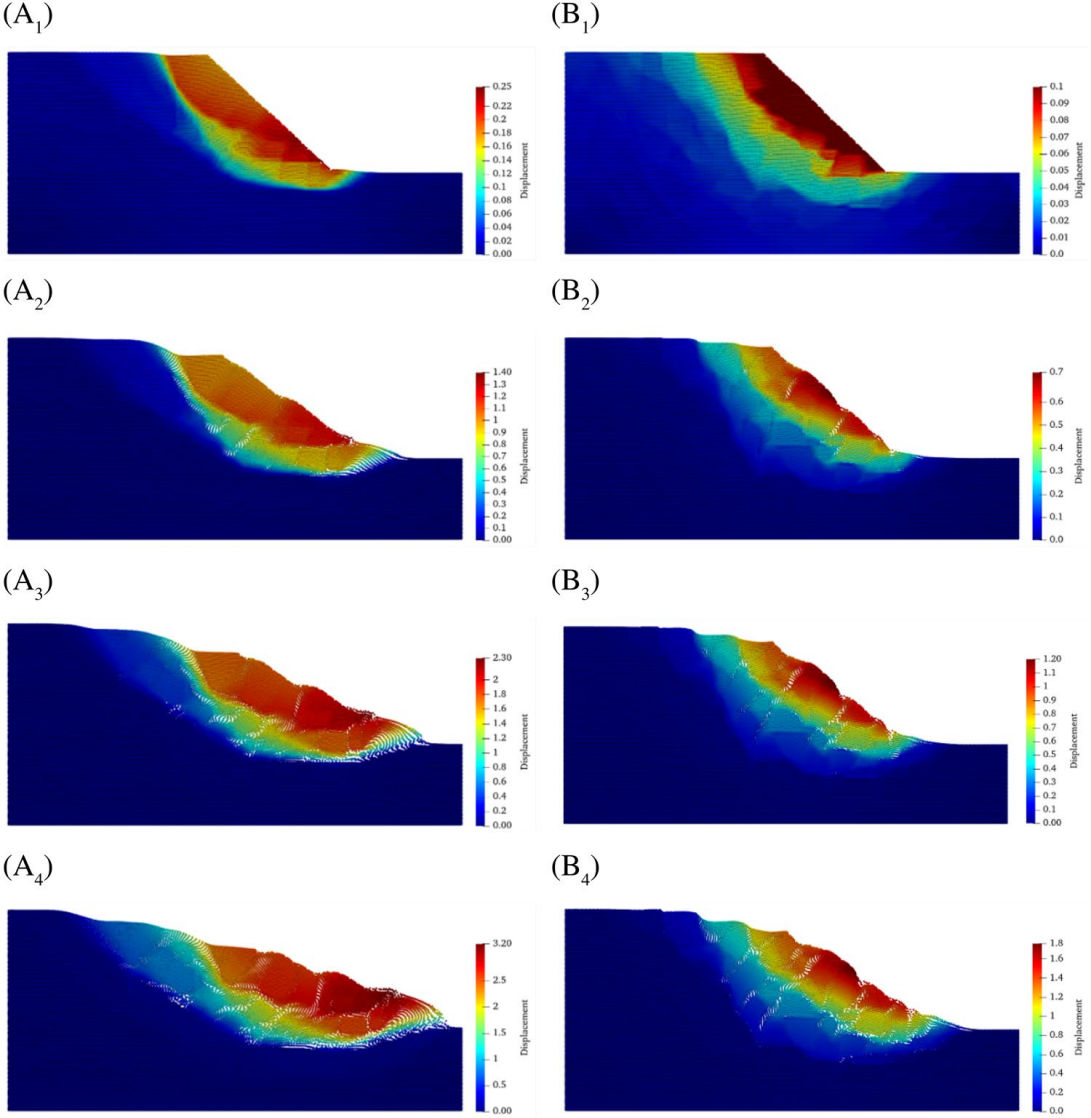
Extension of the displacement of SRM slopes as the discount factor increases. (**A1**–**A4**) Displacement distribution of SRM slope with 10% rock content at the reduction factor equal to 1.9, 2.2, 2.5 and 2.8, respectively. (**B1**–**B4**) Displacement distribution of SRM slope with 20% rock content at the reduction factor equal to 2.4, 2.6, 2.7and 2.8, respectively.

Figure 11 gives the displacement snapshots of the two slope models as the reduction factor increases. Figure 11A1–A4 gives the displacement distributions of the SRM slope with 10% rock content when the discount factor is equal to 1.9, 2.2, 2.5, and 2.8, respectively, and Fig. 11B1–B4 gives the displacement distributions of the SRM slope with 20% rock content when the discount factor is equal to 2.4, 2.6, 2.7 and 2.8, respectively.

From the perspective of displacement distribution, they show the obvious characteristics of stratification, and the maximum displacements are all distributed in the surface layer of the slope. The difference is that the slope with 20% rock has more layers, and the boundary between layers is clear. The slope with 10% rock content has fewer layers, and the boundary lines between layers are smoother and approximate to circular arcs. In terms of displacement values, the displacement of the slope increases significantly with the increase of the reduction factor, and it is noteworthy that the distribution area of the maximum displacement hardly changes at different reduction factors. In other words, the increase of the reduction factor does not change the distribution pattern of the displacements.

### Slope dynamics in extreme conditions

**Figure 12:**
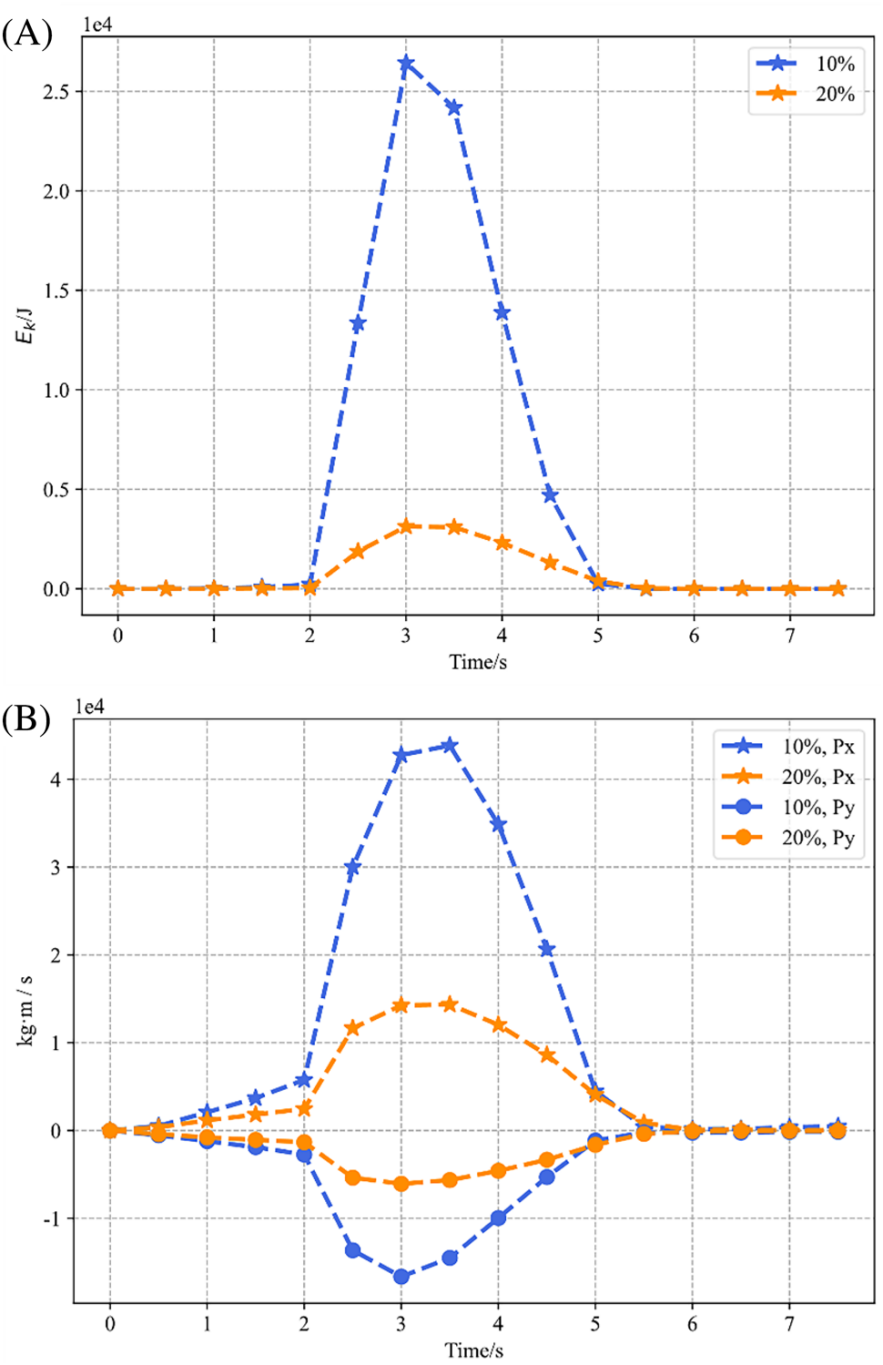
Kinetic energy and momentum variation for SRM slope models with stone content equal to 10% and 20% with the reduction factor equal to 2.8. (**A**) Kinetic energy variations. (**B**) Momentum variations.

**Figure 13:**
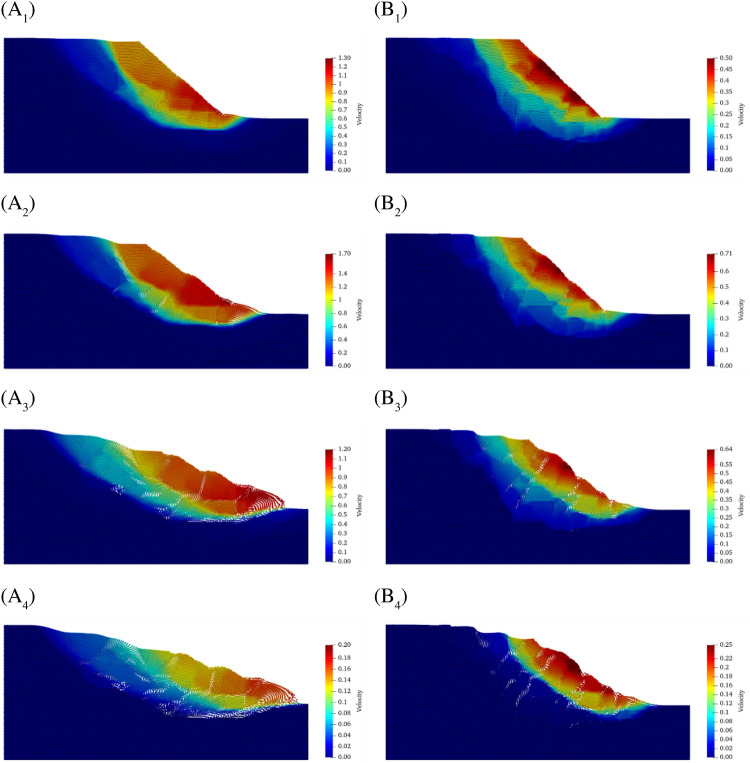
Velocity distribution of SRM slopes for the reduction factor equal to 2.8. (**A1**–**A4**) Velocity distribution of SRM slopes with 10% rock content at t = 2.5 s, 3.0 s, 4 s and 5 s. (**B1**–**B4**) Velocity distribution of SRM slopes with 20% rock content at t = 2.5 s, 3.0 s, 4 s and 5 s.

Both slope models are deformed drastically at the reduction factor equal to 2.8, and this section explores their dynamic characteristics in this working condition. Figure 12 gives the curves of their kinetic energy and momentum changes, and Fig. 13 gives snapshots of their velocities at some typical moments.

According to Fig. 12A, the time of the two model landslides is the same, and both are about 3 s. The slides they experienced can be divided into three phases, namely, the acceleration phase, the deceleration phase, and the stabilization phase. The duration of their acceleration and deceleration phases keeps the same, and they both reach the peak of kinetic energy at 3 s. The difference is that the kinetic energy of the slope with 20% rock content is more moderate, and the peak kinetic energy of the slope with 10% rock content is about five times of the counterpart. According to Fig. 8B, the motion patterns of the two models are still dominated by horizontal motion. However, the kinetic energy of the slope with 10% rock content reaches its peak at the same time as the vertical momentum, which is 0.5 s earlier than the horizontal momentum. The moment when the momentum of the two directions of the counterpart reaches the peak is consistent with the kinetic energy.

Figure 13 gives snapshots of the velocities of the two models during the acceleration and deceleration phases. First, the distribution shows that the velocity distribution has the same characteristics as the displacement distribution, that is, stratified, and the area with the maximum velocity at different moments hardly changes. According to Fig. 13A2,B2, the peak velocity of the slope with 10% rock content is about 2.7 times that of its counterpart. According to the velocity snapshots of the deceleration phase, it is obvious that the slope with 20% stone content decelerates more slowly. At T = 5 s, the maximum velocity of the slope with 20% stone content is 0.25 ms^−1^, while its counterpart is only 0.2 ms^−1^.

## Conclusion

In this study, the author applies the material point strength reduction method to scrutinize the impact of rock content on the stability of Soil–Rock Mixture (SRM) slopes and the kinematics of large displacement landslides post-failure. Our approach involves the use of digital image processing to construct four nuanced material point models, each representing a distinct stone content level to facilitate a comparative analysis.

The core findings from our stability and large deformation analyses are twofold. Primarily, the author demonstrates that an increase in stone content within the SRM substantially improves slope stability. Our models show that when the stone content exceeds 20%, the slopes display remarkable resilience and do not undergo failure, highlighting a possible benchmark for stone content that is critical for ensuring stability.

Secondly, the author observes that the size and distribution of rock content play a significant role in determining the pattern of slope failure. Lower stone contents lead to a nearly circular failure surface, suggesting a more predictable failure mechanism, whereas higher contents result in more complex and less predictable failure patterns.

A notable correlation was also found between the rock content and the dynamic response of the slope after failure. Models with lower stone contents were associated with a more dynamic response, evidenced by a greater release of kinetic energy during the destabilization process. This insight is crucial for risk assessments and designing mitigation strategies for potential landslides.

The incorporation of a GPU-based high-performance Material Point Method (MPM) program into our research has been groundbreaking, significantly enhancing computational efficiency. This improvement has facilitated our current two-dimensional analyses and established a solid foundation for future expansions into three-dimensional modeling, which promises to provide a more comprehensive understanding of SRM slope behaviors and the mechanics of landslides.

In sum, our study sheds light on the significant influence of rock content on SRM slope stability and the post-destabilization dynamics of landslides. The development of advanced computational tools, such as the high-performance MPM program, marks a substantial progression in the field. Looking ahead, the author aims to leverage these tools to advance into three-dimensional simulations, further enriching the geotechnical engineering discipline and contributing to the development of more effective landslide prevention and mitigation measures.

## Data Availability

The datasets used and/or analyzed during the current study available from the corresponding author on reasonable request.
