# Author Correction: Study on large deformation of soil–rock mixed slope based on GPU accelerated material point method

**DOI:** 10.1038/s41598-024-61471-y

**Published:** 2024-05-14

**Authors:** Bingke Liu, Wen Wang, Zhigang Liu, Ningpeng Ouyang, Kejie Mao, Fuchuan Zhou

**Affiliations:** 1 School of Civil Engineering, Chongqing University, Chongqing, 400045 China; 2 Department of Engineering, Durham University, Durham, DH1 3LH UK; 3 China Construction Fourth Engineering Division Corp. LTD, No. 220 Weihai Road, Panyu District, Guangzhou, 511400 China; 4 Hehai College, Chongqing Jiaotong University, Chongqing, 400074 China; 5 Chongqing Vocational Institute of Engineering, Chongqing, 402260 China

Correction to: *Scientific Reports* 10.1038/s41598-024-57362-x, published online 24 March 2024

The original version of this Article contained an error in Reference 10, which was incorrectly given as:

Conte, E., Pugliese, L. & Troncone, A. A simple method for predicting rainfall-induced shallow. *Landslides* **148**, 04022079 (2022).

The correct reference is listed below:

Conte E., Pugliese L. & Troncone A. A simple method for predicting rainfall-induced shallow landslides. *J. Geotech. Geoenviron.* **148**, 04022079 10.1061/(ASCE)GT.1943-5606.0002877 (2022)

The original Article has been corrected.

